# Structural-Functional Changes in a Ti_50_Ni_45_Cu_5_ Alloy Caused by Training Procedures Based on Free-Recovery and Work-Generating Shape Memory Effect

**DOI:** 10.3390/nano12122088

**Published:** 2022-06-17

**Authors:** Mihai Popa, Nicoleta-Monica Lohan, Bogdan Pricop, Nicanor Cimpoeșu, Marieta Porcescu, Radu Ioachim Comăneci, Maria Cazacu, Firuța Borza, Leandru-Gheorghe Bujoreanu

**Affiliations:** 1 Faculty of Materials Science and Engineering, “Gheorghe Asachi” Technical University of Iași, Blvd. Dimitrie Mangeron 71A, 700050 Iași, Romania; mihai.popa@academic.tuiasi.ro (M.P.); nicoleta-monica.lohan@academic.tuiasi.ro (N.-M.L.); bogdan.pricop@academic.tuiasi.ro (B.P.); nicanor.cimpoesu@academic.tuiasi.ro (N.C.); radu-ioachim.comaneci@academic.tuiasi.ro (R.I.C.); 2 National Institute of Research and Development for Technical Physics, Blvd. Dimitrie Mangeron 47, 700050 Iași, Romania; porcescu@phys-iasi.ro (M.P.); fborza@phys-iasi.ro (F.B.); 3 Department of Inorganic Polymers, “Petru Poni” Institute of Macromolecular Chemistry, Aleea Gr. Ghica Voda 41A, 700487 Iași, Romania; mcazacu@icmpp.ro

**Keywords:** TiNiCu SMA, shape memory effect, training, martensite, reverse martensitic transformation, tensile tests, specific enthalpy, storage modulus, phase change, strain sweep

## Abstract

Active elements made of Ti_50_Ni_45_Cu_5_ shape memory alloy (SMA) were martensitic at room temperature (RT) after hot rolling with instant water quenching. These pristine specimens were subjected to two thermomechanical training procedures consisting of (i) free recovery shape memory effect (*FR-SME*) and (ii) work generating shape memory effect (*WG-SME*) under constant stress as well as dynamic bending and RT static tensile testing (*TENS*). The structural-functional changes, caused by the two training procedures as well as *TENS* were investigated by various experimental techniques, including differential scanning calorimetry (*DSC*), dynamic mechanical analysis (*DMA*), X-ray diffraction (*XRD*), and atomic force microscopy (*AFM*). Fragments cut from the active regions of trained specimens or from the elongated gauges of *TENS* specimens were analyzed by *DSC*, *XRD*, and *AFM*. The *DSC* thermograms revealed the shift in critical transformation temperatures and a diminution in specific absorbed enthalpy as an effect of training cycles. The *DMA* thermograms of pristine specimens emphasized a change of storage modulus variation during heating after the application of isothermal dynamical bending at RT. The *XRD* patterns and AMF micrographs disclosed the different evolution of martensite plate variants as an effect of *FR-SME* cycling and of being elongated upon convex surfaces or compressed upon concave surfaces of bent specimens. For illustrative reasons, the evolution of unit cell parameters of B19′ martensite, as a function of the number of cycles of *FR-SME* training, upon concave regions was discussed. *AFM* micrographs emphasized wider and shallower martensite plates on the convex region as compared to the concave one. With increasing the number of *FR-SME* training cycles, plates’ heights decreased by 84–87%. The results suggest that *FR-SME* training caused marked decreases in martensite plate dimensions, which engendered a decrease in specific absorbed enthalpy during martensite reversion.

## 1. Introduction

The equiatomic NiTi shape memory alloy (SMA) was discovered more than sixty years ago at the Naval Ordnance Laboratory, which gave rise to the trade name of NITINOL [1]. Comprehensive research performed over the last six decades has emphasized that the shape memory effect (SME) of NiTi SMA is driven by the thermally induced reversion to B2 austenite of B19′ stress-induced martensite [2]. The crystallographic parameters of the two phases are: (i) *a* = 0.3015 nm for B2 cubic austenite and (ii) *a* = 0.2889 nm, *b* = 0.412 nm, *c* = 0.4622 nm, and β = 96.860 for B19′ monoclinic martensite [3]. Due to the high crystallographic ordering of austenite, the solid-state transition B2 ↔ B19′ represents a reversible thermoelastic martensitic transformation [4].

Consequently, the austenite phase is stiffer than the martensite phase, and the SMA is therefore able to develop work generating (WG) SME during reverse thermoelastic martensitic transformation [5] associated with work outputs exceeding 4 J/g upon heating [6]. This outstanding capacity of NITINOL to develop high specific work output [7] enhanced the development of various applications of work generating SME, such as 3-D manipulators and walking robots [8], robotic hands and clean grippers [9], and biomimetic robots [10]. Recently, SMA-based grippers, end-effectors, and robotic structures for material handling, soft robotics and continuum robotics were developed [11].

Aiming to improve NITINOL’s SM properties, ternary alloys have been investigated mainly by the partial substitution of Ni with the third addition element. Among them, Cu addition up to around 30% caused: (i) narrower transformation hysteresis; (ii) reduction in the dependence of the critical temperature for the start of direct martensitic transformation (M_s_) on concentration variations and cyclic fatigue; (iii) softer martensite phase requiring lower reset forces in cyclic applications; (iv) two-step martensitic transformation and corresponding two-step shape change (at 7.5–20% Cu) [12].

The two-step reversible thermoelastic martensitic transformation occurs in TiNiCu SMAs, especially if Cu amounts exceed 7 at.%, according to the sequence: B2 cubic austenite ↔ B19 orthorhombic martensite ↔ B19′ monoclinic martensite [13]. B19 orthorhombic martensite is considered an intermediate phase [14]—its formation typically occurring after solution treatment and short-time ageing, especially if the SMA contained more than 7 at.% Cu. When it occurred at 5.5 at.% Cu, it was considered unstable [15].

In order to develop applications with repetitive *WG-SME*, a two-way shape memory effect (TWSME) must be obtained by means of a special thermomechanical treatment called “training”, after which an SMA part would spontaneously recover both its hot and cold shapes during heating and cooling, respectively [16].

At SMAs, thermomechanical training basically comprises the repeated passage along a prescribed path in the strain–stress–temperature space until a closed loop is reached. Consequently, a large variety of training procedures have been reported based on the cyclic transformation from austenite to preferentially oriented martensite [7].

In the general case of TiNiCu SMAs, various training combinations were tested, such as ageing and superelastic compression [17], pre-straining in the martensite state [18], or an alternation of cooling enhanced extension and heating-triggered contraction of helical springs [19].

In the particular case of Ti_50_Ni_45_Cu_5_ SMAs, where the formation of B19 martensite was not reported, deformations were applied to coil springs, causing recovery rate increases up to a critical deformation and the decrease in critical temperatures for the start and finish of the reverse martensitic transformation, A_s_ and A_f_, respectively [20].

Among the training procedures applied to SMAS with a thermoelastic martensitic transformation, there is a free-recovery shape memory effect (*FR-SME*) and a work-generating shape memory effect (*WG-SME*). The former comprises repeated deformation-heating–cooling cycles, but, as some of the present authors previously reported in the case of a Cu-Zn-Al SMA, amnesia (memory loss) may occur if a long period of time elapses between consecutive complex cycles [21]. The latter consists of the heating-induced lifting of a load fastened at the free end of a martensitic specimen, which becomes austenitic, followed by the cooling-induced lowering of the load due to martensitic softening.

Therefore, the present study aims to analyze the effects of two training procedures—*FR-SME* and *WG-SME*—on the structure and properties of a Ti_50_Ni_45_Cu_5_ SMA. The former will be applied for longer time periods, reaching up to two weeks, and the evolution of the reverse martensitic transformation will be analyzed. The latter will be applied with progressively increasing loads up to the movement-generation limit of Ti_50_Ni_45_Cu_5_ SMA.

## 2. Materials and Methods

A Ti_50_Ni_45_Cu_5_ SMA was produced from: (i) metallic Titanium, Grade 1 ASTM B381-13 (2019), with 0.20% Fe, 0.03% N_2_, 0.18% O_2_, 0.015% H_2_, 0.08% C, and balance Ti; (ii) metallic Nickel (cathode), ASTM A494 GR CZ-100, with min. 99.95% Ni; and (iii) electrolytic Copper, SR ISO 431-1995, with 99.99% Cu. Raw materials were: (i) cut with a mechanical saw into pieces smaller than 10 mm × 5 mm × 5 mm; (ii) cleaned in an ultrasonic bath; (iii) dried; and (iv) degreased with acetone. Then, the three elements were loaded into the fusion crucible of a FIVE CELES (Lautenbach, France) levitation induction furnace in the increasing order of melting points. After producing advanced vacuum (10^−4^ mm Hg) to expel all the gases, low-pressure (−0.2 … −0.3 bar) Ar was introduced to avoid oxidation and to reduce evaporation losses. Melting was carried out at a frequency of 106 kHz with a power consumption of 22 kW. The melt was cast into water-chilled 20 mm-diameter copper forms. The ingots were machined into 18 mm-diameter cylinders with a length of 40 mm. By means of a DEM 320 wire sparking machine, the ingots were cut into 2 mm-thick longitudinal slices, which were hot-rolled at 1000–1050 °C with an experimental roller. Then, they were instantly water quenched after leaving the rolling cylinders [22]. The final hot rolling thickness obtained after several calibration passes was 0.5 mm. By wire spark erosion, specimens with various geometries were cut for tensile tests (*TENS*), dynamic mechanical analysis (*DMA*), as well as *FR-SME* and *WG-SME* training.

*TENS* specimens had two geometries designated as *opm* (2 mm × 3 mm gauge) and *OPM* (4 × 20 mm gauge). An INSTRON 3382 (Norwood, MA, USA) tensile machine and a cross-head speed of 1 mm/min were used to subject two specimens of both *opm* and *OPM* geometries to a failure test and four loading–unloading tests to 300 MPa stress.

From various specimens in different states, five fragments weighing less than 50 mg were cut with a MIRACUT 150 precision cutter and were subjected to differential scanning calorimetry analysis (*DSC*) by means of a NETZSCH *DSC* 200 F3 Maia (Selb, Germany) differential scanning calorimeter after water flow grinding to remove superficial corrosion. The device, with a sensitivity <1 W, a temperature accuracy of 0.1 °C, and an enthalpy accuracy of, generally, <1%, was calibrated with Bi, In, Sn, and Zn standards and used correction curves. Fragments cut from the tensile broken/pre-strained specimens were analyzed by *DSC* on Setaram Labsys equipment (SETARAM, Plan-les-Ouates, Switzerland). The experiments were performed under a protective Ar atmosphere and liquid nitrogen cooling, with a temperature variation rate of 10 °C/min. The tests performed on the *DSC* 200 F3 Maia also used a heating rate of 5 °C/min.

*DMA* specimens were 0.5 mm × 4 mm × 50 mm and were tested on a NETZSCH *DMA* 242 Artemis device with a dual cantilever specimen holder using a frequency of 1 Hz. Two types of tests were performed: (i) temperature scans heating up to 400 °C, with a heating rate of 5 °C/min and a maximum amplitude of 166 µm, and (ii) strain sweeps at room temperature (RT) over five cycles with amplitudes increasing from 1 to 100 µm.

**Figure 1:**
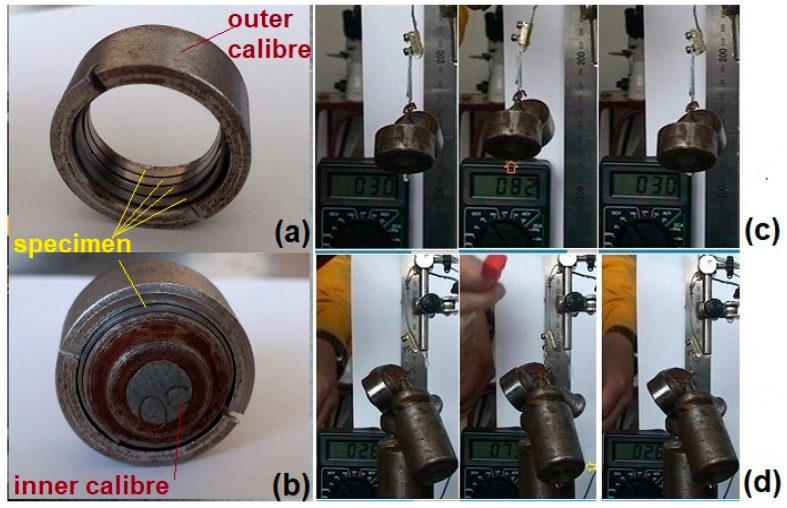
Experimental training procedures based on: (**a**) *FR-SME* with the outer caliber cold shape setting; (**b**) *FR-SME* with the outer and inner caliber cold shape setting; (**c**) *WG-SME* under a 170 g applied load; (**d**) *WG-SME* under a 950 g applied load.

For *FR-SME* and *WG-SME* training, several 0.5 mm × 2 mm × 60 mm active elements were cut by wire spark erosion. The elements—weighing 0.44 g each—were subjected to the experimental tests illustrated in Figure 1.

For *FR-SME* training, five active elements were first introduced to an outer caliber according to Figure 1a and then additionally fixed with an inner caliber, as in Figure 1b. Then, each of the five elements was released after one day and heated with a hot-air gun. Once the maximum possible shape recovery was reached, the elements were water-cooled before being fixed again between calibers. All these actions represent one cycle. One active element was retained after 2 days/cycles, and the remaining three were further cycled. Thus, the second element was retained after 4 days/cycles, the third after 8 days/cycles, and the fourth after 16 days/cycles. Therefore, in the end, four groups of five active elements each have undergone 2, 4, 8, and 16 cycles of *FR-SME* training, respectively.

*WG-SME* training comprised a series of ten heating–cooling cycles with increasing loads, between 100 and 1500 g, fastened at the free end of an active element. In the case of a load of 100, one specimen was separately trained for 50 cycles. Figure 1c shows the specimen’s positions at 30 °C, 82 °C, and again at 30 °C. The orange arrow marks the load’s lifting during the heating up to 82 °C. When the applied load increased, vertical lifting became less obvious, yet a horizontal displacement could be noticed, as marked with a yellow arrow, at 73 °C for an applied load of 950 g, as shown in Figure 1d.

Fragments cut from trained or elongated specimens were analyzed by X-ray diffraction (*XRD*) by means of a D8 Advance Bruker diffractometer with CuKα monochromatized radiation (Kα = 1.5418 Å), a 2θ measuring range between 25 and 85°, and a measuring step of 0.02/4 s. A quantitative and qualitative evaluation was performed using the ICDD database PDF 2 Release, 2016, corresponding to the theoretical crystallographic phase, using Diffrac EVA software. The following ICDD databases were used: (i) 00-052-0860 for B2 cubic austenite (Fm-3m space group); (ii) 00-035-1281 for B19′ monoclinic martensite (P2_1_/m); (iii) 00-044-0113 for TiNi_0.8_Cu_0.2_ orthorhombic martensite (Cmcm); (iv) 00-039-1113 for Ni_4_Ti_3_ rhombohedral (R-3); (v) 00-018-0460 for Cu_4_Ti_3_ (I4/mmm); and (vi) 01-071-5576 for TiO_2_, tetragonal (P4_2_/mnm). The Powley fitting was used to calculate/refine the lattice parameter for the main phase, B 19′ (monoclinic), with the profile factors Rp and Rwp being smaller than 11.65 for Rp and smaller than 13.05 for Rwp. The Goodness of Fit (GOF) is smaller than 1.21.

In order to observe the morphologic particularities of martensite plates, atomic force microscopy (*AFM*) micrographs were recorded on electropolished surfaces using a NanoSurf (Liestal, Switzerland) easyScan 2 device equipped with silicon SPM cantilevers and an easyScope video camera at a scanning rate of 2 × 10^−1^ s per line.

## 3. Results and Discussion

### 3.1. Evolution of Specimens’ Profile during Training

**Figure 2:**
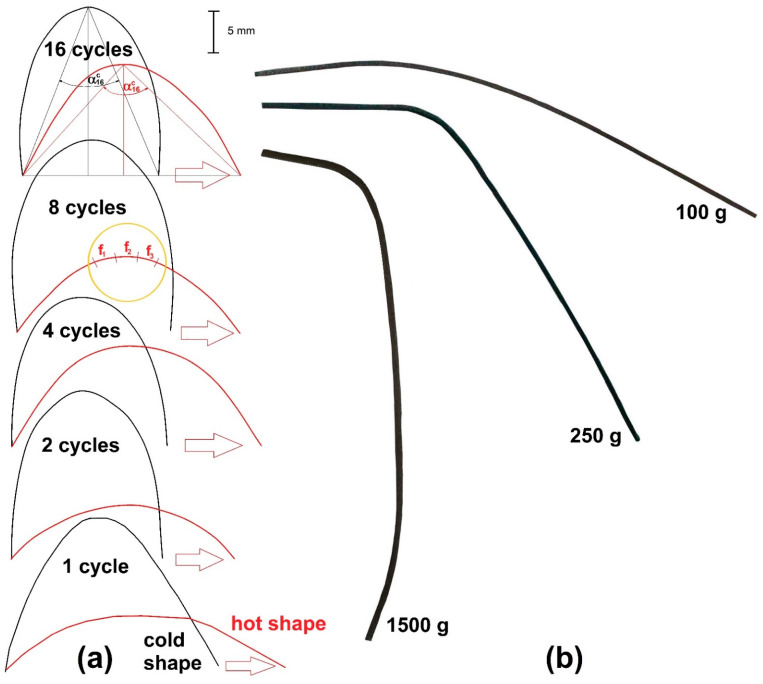
Evolution of specimens’ profiles during experimental training procedures based on: (**a**) *FR-SME* during 16 cycles; (**b**) *WG-SME* under applied loads, increasing up to 1500 g.

The specimens’ profiles after final cooling were recorded after *FR-SME* and *WG-SME* training, as summarized in Figure 2.

Figure 2a illustrates the profiles of cold shapes obtained after the specimens were released from calibers and the hot shapes after hot-air gun heating and water cooling. It is noticeable that with an increase in the number of cycles, the specimens became more and more deformed, in such a way that even the two ends of the specimens’ cold shape became curved, in spite of the inherent elastic spring back that should normally occur after specimens’ release from calibers. On the other hand, the hot shapes also remained more and more deformed as compared to the initial straight form, but this could be an effect of TWSME, which triggered specimens’ deformation during final cooling. As illustrated in Figure 2a of the specimen trained for 16 cycles, the average deflection angles were measured both for cold (*α^c^*) and hot (*α^h^*) shapes. The values of the two angles for each of the profiles shown in Figure 2a are summarized in Table 1.

In Table 1, *SRD* represents the shape recovery degree calculated with Equation (1):

*SRD* = (*α^h^* − *α^c^*)/(180 − *α^c^*) × 100 (1)

where 180° corresponds to the ideal straight hot shape of the specimen in its initial state.

Thus, it appears that the *FR-SME* training procedure contributed to the decrease of *SRD,* probably due to martensite stabilization and implicitly to the diminution of reverse martensitic transformation, as will be demonstrated later.

Figure 2b displays the profiles of the specimens trained by *WG-SME*. Obviously, the specimens became more deformed under larger applied loads. In the case of a 1500 g load, the trained specimen acquired two curvatures—one caused by the load’s weight and the other by its lateral displacement—since the specimen was unable to lift the load. It is expected that deformation enhancement, alongside increasing the mass of the lifted weight during *WG-SME* cycles, would normally increase the amount of stress-induced martensite.

### 3.2. Tensile Testing (TENS)

**Figure 3:**
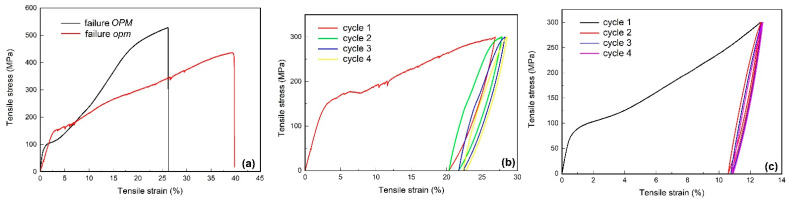
Tensile stress–strain curves: (**a**) failure of *opm* and *OPM* specimens; (**b**) four loading–unloading cycles, *opm* specimen, up to 300 MPa; (**c**) four loading–unloading cycles, *OPM* specimen, up to 300 MPa.

The results of *TENS* are summarized in Figure 3.

It is well-known that tensile properties strongly depend on geometry, especially in the case of polycrystalline specimens, where deformation can be strongly impeded by intergranular incompatibilities [23]. For this reason, the two failure curves from Figure 3a display different slopes in the elastic regions, and the loading–unloading curves have different stress plateaus as well as different total and permanent strains. Based on the total (*ε_t_*), permanent (*ε_p_*), and remanent (*ε_rem_*) strains [24], as well as on dissipated (*E*_1_) and unloading-released (*E*_2_) energies, proportional to the surface areas between loading–unloading curves and under unloading curve, respectively, [25] the energy storage efficiency (*η*) and specific damping capacity (*SDC*) were calculated with Equations (2) and (3), respectively:

*η* = *E*_2_/(*E*_1_ + *E*_2_) × 100 (2)

*SRD* = *E*_1_/(*E*_1_ + *E*_2_) × 100 (3)

**Figure 4:**
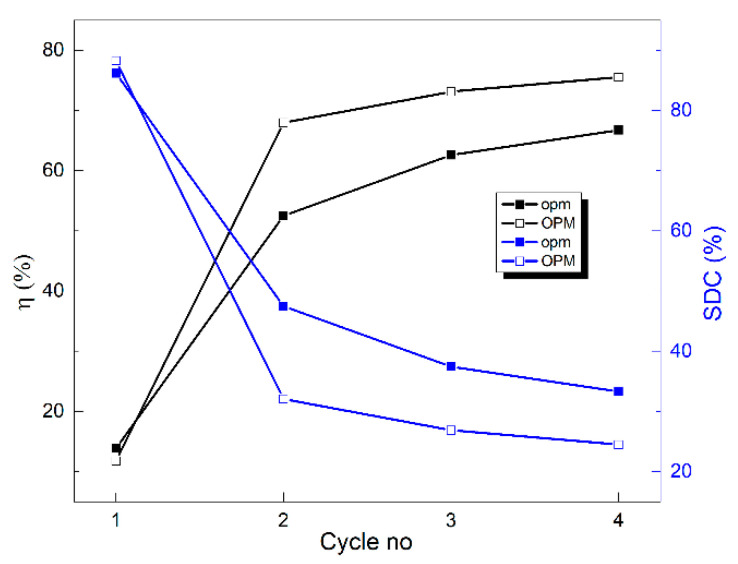
Evolution of energy-storage efficiency (*η*) and specific damping capacity (*SDC*) with the number of loading–unloading cycles according to Figure 3b,c.

The variations in the two parameters with the number of tensile loading–unloading cycles are illustrated in Figure 4.

During *TENS*, the Ti_50_Ni_45_Cu_5_ specimens accumulated more and more energy, which they released during unloading, thus causing the increase in energy storage efficiency *η*. On the other hand, *SDC* decreased with the increase in the number of tensile cycles, which means that internal friction also decreased, thus suggesting a diminution in interface mobility during the reversible formation of stress-induced martensite [26].

### 3.3. Differential Scanning Calorimetry (DSC)

**Figure 5:**
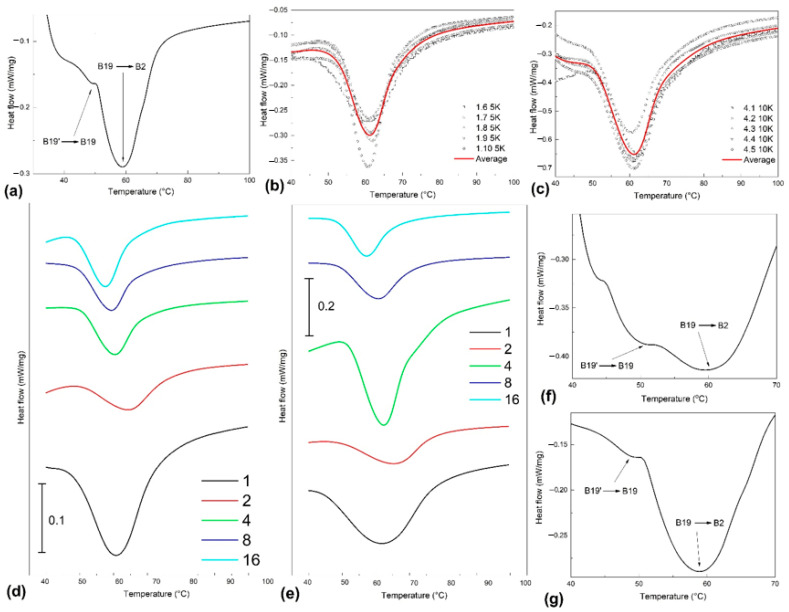
*DSC* thermograms of fragments cut from specimens trained by *FR-SME* during up to 16 cycles at different heating rates: (**a**) initial hot-rolled water-quenched state; (**b**) experimental and average heat flow values at a heating rate of 5 °C/min for a specimen trained for 1 cycle; (**c**) experimental and average values at 10 °C/min for a specimen trained for 16 cycles; (**d**) average values for specimens heated at 5 °C/min; (**e**) average values for specimens heated at 10 °C/min; (**f**) detail of average heat flow values for a specimen trained for 8 cycles heated at 1 °C/min; (**g**) detail of average heat flow values for a specimen trained 16 cycles heated at 1 °C/min.

The first series of *DSC* thermograms emphasize the effects of *FR-SME* training on the thermodynamic behavior of the specimen in initial hot-rolled and water-quenched conditions, subjected to heating, as summarized in Figure 5. In this case, only the heating part of the cycle was displayed because *FR-SME* represents the unique recovery of a hot shape during the heating of an SMA to whom a cold shape was induced [2].

Figure 5a shows two overlapping endothermic peaks that may be associated with a two-step reverse martensitic transformation. Figure 5b,c display with symbols the experimental heat flow values recorded for specimens trained for 1 and 16 cycles, respectively, while the average variations are illustrated with solid lines. It is obvious that the thermal response recorded at 10 °C/min is much stronger than that obtained at 5 °C/min. The average heat flow variations are shown in Figure 5d,e for heating rates of 5 and 10 °C/min, respectively. The larger width of the experimental endothermic peaks, as compared to those found in the literature [27], suggests the presence of two-step reverse martensitic transformation, as confirmed by Figure 5f,g, recorded at 1 °C/min heating rate, on specimens subjected to 8 and 16 *FR-SME* training cycles, respectively. The specific absorbed enthalpies (Δ*h_endo_*) and the peak temperatures (*A*_50_) were evaluated on the ten endothermic peaks from Figure 5d,e, according to Table 2.

The following evolution tendencies can be noticed:

- Heating rate increasing, from 5 to 10 °C/min, caused higher values for both Δ*h_endo_* (in absolute value) and *A*_50_, in good agreement with previous results reported by some of the present authors, in the case of a Cu-Zn-Al SMA [28];
- *A*_50_ values had a general decreasing tendency with increasing the number of cycles;
- Δ*h_endo_* values have a general decreasing trend (in absolute values) with the increase in the number of training cycles.

The above variation tendencies suggest that the initial B19 orthorhombic was replaced by B19′ monoclinic martensite, which reverted to austenite during heating, decreasing in proportion with the increase in the number of *FR-SME* training cycles, but became less stabilized since it required less thermal energy support to trigger the transformation. Concerning the discrepant variation of absorbed enthalpy observed after four cycles, it could be explained by the mechanism proposed by Kajiwara and Kikuchi. They observed that dislocations were accumulated along martensite–austenite interface and were periodically released in the matrix. The observed increase in the amount of absorbed enthalpy during the first cycles of martensite reversion could be an effect of dislocation trapping along the austenite–martensite interface during its repetitive movement from austenite to martensite. After several passages of the interface, emissary dislocations were emitted, the matrix remained freer and freer of dislocations, and, thus, the interface passage required less energetic consumption [29].

**Figure 6:**
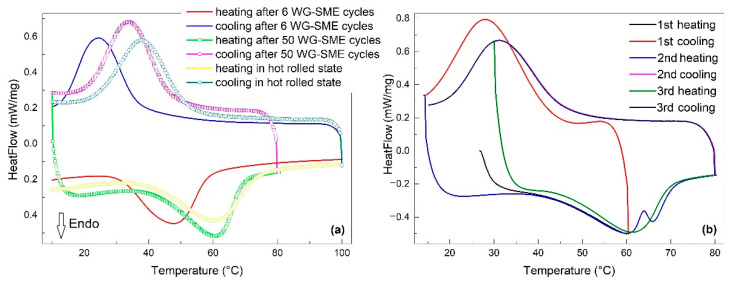
Heating–cooling *DSC* thermograms of fragments cut from specimens trained by *WG-SME*: (**a**) after different numbers of cycles; (**b**) temperature memory effect after 50 training cycles with 100 g load.

The second series of *DSC* thermograms were recorded during the heating and cooling at 10 °C/min of fragments cut from specimens trained by *WG-SME* and are summarized in Figure 6. Both heating and cooling parts of the *DSC* charts were displayed because *WG-SME* training does not require operator intervention to induce the cold shape after cooling, as in the case of *FR-SME.*

In this case, again, the evaluation of the areas of endothermic and exothermic peaks enabled determining the specific absorbed (Δ*h_endo_*) and released (Δ*h_exo_*) enthalpies as well as the peak temperatures of reverse (*A*_50_*)* and direct *(M*_50_*)* martensitic transformations, which are listed in Table 3.

When comparing the Δ*h_endo_* and *A*_50_ values of the specimens in the initial state and after 50 training cycles from Table 3 with those from Table 2, it is obvious that *WG-SME* training cycles have preserved both the specific absorbed enthalpy and peak temperature values from the first *FR-SME* cycles. This observation suggests that *WG-SME* training has been more beneficial to the martensitic transformation as compared to *FR-SME*. After *WG-SME* training, no decreasing tendencies were noticed both at Δ*h_endo_* and *A*_50_. In addition, the reversible martensitic transformation that occurred at the specimen trained for 50 *WG-SME* cycles under a 100 g load has been fully reproducible for three heating–cooling cycles. Moreover, the temperature memory effect (TME) has been emphasized in Figure 6b after interrupting the first heating at 60 °C. TME occurred in the second heating under a characteristic form with two endothermic peaks. As expected, the interruption of the second cooling cycle did not cause any peak splitting [30].

**Figure 7:**
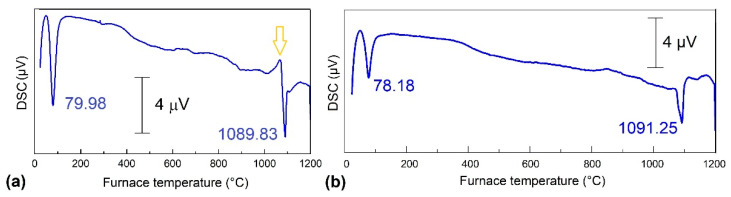
*DSC* thermograms of fragments cut from tensile-tested *opm* specimens: (**a**) after failure, according to Figure 3a; (**b**) after four loading–unloading cycles, up to 300 MPa, according to Figure 3b.

Aiming to observe the eventual solid-state transitions that might occur during heating above the thermal range for reverse martensitic transformation, *DSC* experiments were performed up to 1200 °C on fragments cut from the *opm* specimens subjected to tensile tests, according to Figure 3. The results are summarized in Figure 7.

The thermogram from Figure 7a was recorded on two fragments cut from each of the two ends of the broken *opm* specimen that failed, according to Figure 3a. The first endothermic peak, located at *A*_50_ = 79.98 °C, can be associated with reverse martensitic transformation and the second, located at 1089.83 °C, with melting. It is noticeable that martensite reversion absorbed more energy than melting since the area of the former peak is larger than the area of the latter.

The yellow arrow marks an exothermic peak that might correspond to recrystallization [31], which is expected to occur after applying a permanent strain of 39.24%, observed after failure at 436 MPa, as shown in Figure 3a. At the end of the experiment, the two fragments became welded.

Figure 7b corresponds to a fragment of the specimen’s gauge, which was permanently elongated by 22.6% at 300 MPa, according to Figure 3b. In this case, martensite reversion occurred at a lower temperature, and no obvious exothermic peak that could be associated with recrystallization was noticeable, probably due to the lower amount of permanent strain.

*A*_50_ temperature was higher at broken specimens—79.98 °C—as compared to the specimen subjected to four loading–unloading cycles—78.18 °C—which, in turn, was higher than the value determined for the specimen subjected to 50 *WG-SME* training cycles under a 100 g load, 60.5 °C. In this case, maximum bending stress can be determined with:

(4) $$\sigma_{\max}=\frac{M_{z}}{W_{z}}$$

where *M_z_* is the bending moment, which equals *mgl* (*m* = load’s mass, 0.1 kg; *g* = gravitational acceleration 9.81 ms^−2^; and *l* = specimen’s length, 0.06 m), and *W_z_* is the resistance modulus = *bh*^2^/6 (*b* = specimen’s breadth, 0.002 m, and *h* = specimen’s thickness, 0.0005 m). After the substitution of all these values in Equation (4) and the conversion of Pa (1 N/m^2^) into MPa, it follows that *σ_max_* ≈ 0.7 MPa.

Therefore, the decrease in *A*_50_ values (79.98 °C at 436 MPa, 78.18 °C at 300MPa, and 60.5 °C at 0.7 MPa) could be correlated with Clausius–Clapeyron equation:

(5) $$\frac{d\sigma_{cr}\left(T\right)}{dT}=\frac{\Delta S}{\varepsilon_{0}}$$

where *σ_cr_(T)* is the critical stress, Δ*S* is the entropy change, and *ε*_0_ is the transformation strain [32].

### 3.4. Dynamic Mechanical Analysis (DMA)

**Figure 8:**
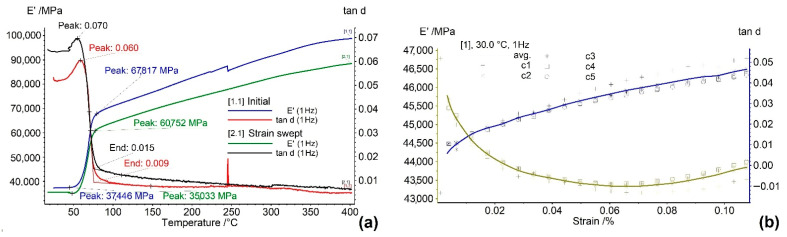
*DMA* thermograms illustrating the variations in the storage modulus (*E’*) and internal friction (*tanδ*) of hot-rolled specimens as a function of (**a**) temperature during temperature scans in the initial state and after an isothermal strain sweep at RT; (**b**) strain amplitude during strain sweep at RT.

*DMA* experiments aimed to emphasize the variations in the storage modulus, *E’,* and internal friction, *tanδ = E”/E’* (*E”*—loss modulus), during forced dynamic bending with a frequency of 1 Hz during heating (temperature scan) at a constant amplitude (166 μm) or during isothermal (RT) amplitude increase from 1 to 100 μm (strain sweep). After subjecting the first specimen to a temperature scan, the second was first subjected to a strain sweep and then to a temperature scan. The results are summarized in Figure 8.

The variations in *E’* and *tanδ* of the specimen in an initial state, as shown in Figure 8a, are in good concordance with the results found in literature for Ti_50_Ni_45_Cu_5_ SMA. The “extraordinarily high peak” [33] of the internal friction maximum reached 0.06 at approx. 60 °C and can be associated with a reverse martensitic transformation [34].

This temperature coincides with *A*_50_ values determined by *DSC* from Figure 5 and Figure 6. Concomitant with the *tanδ* maximum, there is a sharp increase in the storage modulus, which is a characteristic feature of a thermoelastic martensitic transformation [35]. The storage modulus increased from 37,446 MPa at 45 °C to 67,817 GPa at 79 °C, which gives a hardening rate of approx. 893 MPa/°C. This storage modulus variation rate is more than four times larger than the value found in the literature for a Ti_50_Ni_45_Cu_5_ SMA: 210 MPa/°C [36]. This result could be the effect of five cycles of RT reversive bending performed at 1 Hz with increasing amplitudes from 1 to 100 µm. *E’* continued to increase during heating, reaching almost 100 GPa. After the reverse martensitic transformation, *tanδ* dropped to about 0.01 because B2 austenite has a smaller damping capacity due to the dynamic/static hysteresis of lattice defects [37].

During five cycles of isothermal strain sweeps at RT, the specimen was completely martensitic, and *tanδ* variations were caused by the stress-induced motion of the interfaces between martensite plate variants [38]. Figure 8b illustrates an increase in *tanδ* with the increase in strain amplitude, which was explained by the intensification of the twin boundary’s movement to accommodate increasing strains [39]. This twin boundary movement intensification can be the cause of the decrease in the storage modulus during the increase in the strain amplitude, as shown in Figure 8b. When the strain-swept specimen was subjected to a new temperature scan during heating, it is expected that the storage modulus values will be lower and internal friction ones will be higher as compared to the values found with the initial specimen, as is the case in Figure 8a, for the entire temperature range.

### 3.5. X-ray Diffraction (XRD)

Three 6 mm-long fragments were cut from each specimen trained by *FR-SME* and were stuck side-by-side on an adhesive paper, in such a way as to form a 6 × 6 mm square, before being analyzed by *XRD*. Figure 2a highlights the sections where the three fragments (f_1,2,3_) were cut from the specimen and subjected to eight *FR-SME* training cycles. 

**Figure 9:**
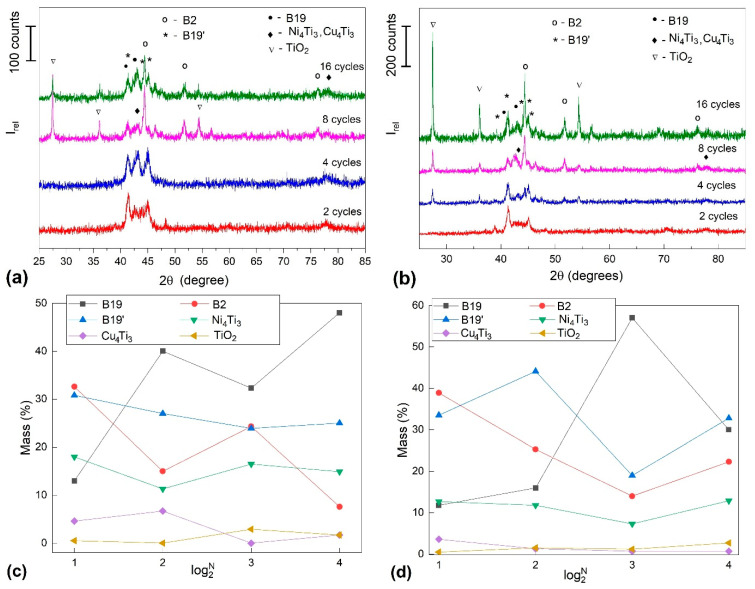
*XRD* patterns of specimens trained for N cycles by *FR-SME*: (**a**) on convex surfaces; (**b**) on concave surfaces; (**c**) semi-quantitative analysis for convex surfaces; (**d**) semi-quantitative analysis for concave surfaces.

Analyses were performed on different fragment groups, exposing convex and concave surfaces. The first series of representative results are shown in Figure 9 for *FR-SME*-trained specimens.

According to the crystallographic databases mentioned in Section 2, six metallographic phases can be identified: (i) B2-cubic austenite, (ii) B19′ monoclinic martensite, (iii) B19 orthorhombic martensite, (iv) Ni_4_Ti_3_ precipitates, (v) Cu_4_Ti_3_ compounds, and (vi) TiO_2_ oxide. The positions of the *XRD* peaks demonstrate that, from a qualitative point of view, there are no differences between the phases present on convex (Figure 9a) or concave (Figure 9b) surfaces. What differs are their relative amounts, as illustrated by semi-quantitative analysis performed by comparing the heights of *XRD* peaks instead of their areas, as shown in Figure 9c,d, respectively.

It is noticeable that the TiO_2_ rutile phase is mostly emphasized by the peak located at 2θ < 30° and is more prominent in the specimens subjected to a large number of cycles. Considering the high oxidation tendency of Ti, these increases in the amount of TiO_2_ oxide were disregarded in the semi-quantitative analysis of the specimens subjected to eight and sixteen *FR-SME* training cycles. These specimens were additionally heated at least four and twelve times, respectively, as compared to the rest of the specimens. For this reason, the quantity of TiO_2_ did not exceed 3 mass.%, while that of Cu_4_Ti_3_ is below 6.7%. From a quantitative point of view, the next phase is represented by Ni_4_Ti_3_ precipitates that occupy a maximum of 18.2% on the convex surface after the first *FR-SME* training cycle. It has been argued that their formation depletes the surrounding matrix in Ni, thus enhancing the superelastic response [40].

The main metallographic phases are B2 austenite and B19′ and B19 martensites, which together occupy more than 70%. B2 austenite can be identified using its representative diffraction peaks (100)_B2_, (110)_B2_, (200)_B2_, and (211)_B2_ [41]. The coexistence of B19′ and B19 martensites at RT was reported in several studies, although it has been demonstrated that B19 orthorhombic martensite can form in Ti_50_Ni_45_Cu_5_ SMAs, but it is instantly replaced by B19′ monoclinic martensite [5]. According to 00-035-1281 and 00-044-0113 databases, the main diffraction maxima are (1$\bar{1}$1)_B19′_, located at 2θ = 41,365°, and (020)_B19_, located at 2θ = 42,675°, respectively, which makes it rather difficult to distinguish between the peaks of the two martensitic phases [42]. Nevertheless, with the increase in Cu content, stable B19 orthorhombic martensite was obtained together with B19′ monoclinic martensite. For instance, B19 and B19′ martensites were identified in the *XRD* patterns of a Ti_50_Ni_44.5_Cu_5.5_ SMA solution treated at 800 °C/1h/water-quenched (W.Q.) and aged (150 °C/24 h) [15] and of a Ti_50_Ni_44_Cu_6_ SMA solution treated at 900 °C/2h/W.Q. [43]. Moreover, above 10 at% Cu, B19 martensite has become the predominant martensitic phase [44].

According to Figure 9c,d, B19 martensite became the prominent phase after 8 *FR-SME* training cycles, both on convex and concave surfaces, reaching a maximum of 57% on the concave surface. On both surfaces, with increasing the number of training cycles (*N*), general decreasing tendencies are noticeable for both B2 austenite and B19′ martensite, while the amount of B19 orthorhombic martensite tends to increase.

The different phase amounts obtained on convex and concave surfaces may be the effect of different loading modes during repetitive bending, as previously observed at Cu-Zn-Al SMAs [45]. It has been argued that, due to the elongation of convex surfaces and the compression of concave ones, martensite plates acquired different morphologies in the two areas: wide and shallow on the former and thin and deep on the latter [46].

**Figure 10:**
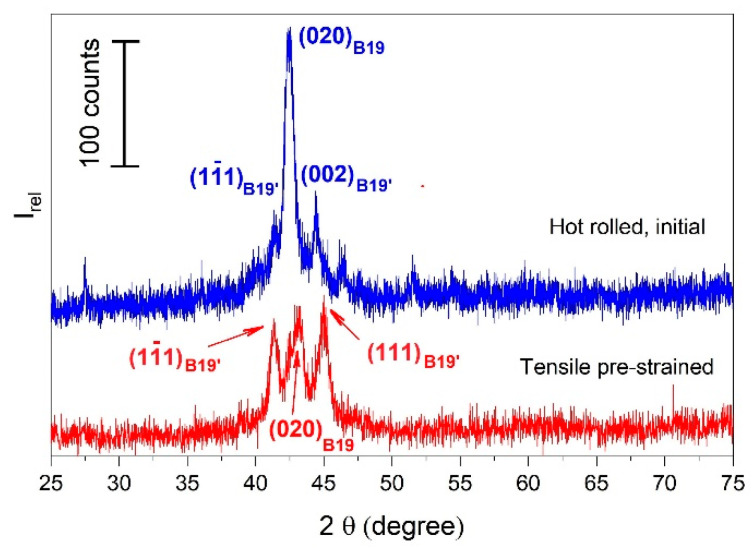
*XRD* patterns of specimens initial and elongated states, according to Figure 3c.

The effects of subjecting the *OPM* specimens to a permanent strain of 11%, at RT, according to Figure 3c, are illustrated by the *XRD* patterns from Figure 10.

The *XRD* pattern of the initial hot-rolled water-quenched state comprises both B19 orthorhombic and B19′ monoclinic martensites, which might sustain the two-step transformation, as illustrated in Figure 5a. After permanent straining at 11%, the two B19′ martensite plate variants were modified in such a way that the amount of (1$\bar{1}$1)_B19′_ increased while (002)_B19′_ was reoriented to (111)_B19′_. It can be assumed that RT tensile straining enhanced the formation of B19′ monoclinic martensite on behalf of B19 orthorhombic since the main diffraction maximum of the former increased in intensity, while that of the latter decreased [47].

**Figure 11:**
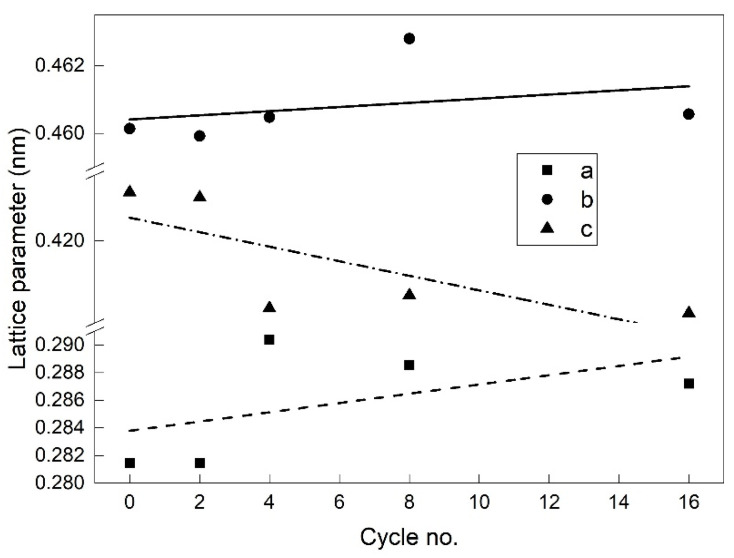
Variation in unit cell parameters of B19′ monoclinic martensite determined with the *XRD* patterns from Figure 9b recorded on concave surfaces as a function of the number of *FR-SME* training cycles. Experimental values with symbols and linear fits with lines.

From Figure 9 and Figure 10, it can be noticed that the 2θ values of the *XRD* peaks corresponding to some metallographic phases have been shifted both by training cycles and by tensile straining [48]. A comparison of the values of the two 2θ shifts caused by training and tensile straining is beyond the scope of the present paper. This shift originates from a change in unit cell parameters, which may vary with temperature and chemical composition [49]. By means of the Rietveld method/Topas program, unit cell parameters were calculated for the main crystallographic phases. The results obtained for the parameters of the unit cell of B19′ monoclinic martensite, observed on the concave surfaces according to Figure 9b, are exemplified in Figure 11.

It appears that the parameters *a* and *b* tend to increase, while *c* tends to decrease with increasing the number of *FR-SME* training cycles. Linear fits were performed according to:

*y* = *I* + *s* × *N* (6)

where *y* is the lattice parameter, *I* is the lattice parameter before cycling, and *s* is the lattice parameter variation rate with the number of cycles (*N*). The linear fit summary is listed in Table 4.

The data found in Table 4 demonstrate that the above-mentioned variation tendencies of lattice parameters are correct. The maximum increasing rate of approx. 7 × 10^−4^ nm/cycle occurred at parameter *a*, while the maximum decreasing rate of approx. −13 × 10^−4^ nm/cycle occurred at parameter *c*, with the increase in the number of *FR-SME* cycles.

### 3.6. AFM Observations

**Figure 12:**
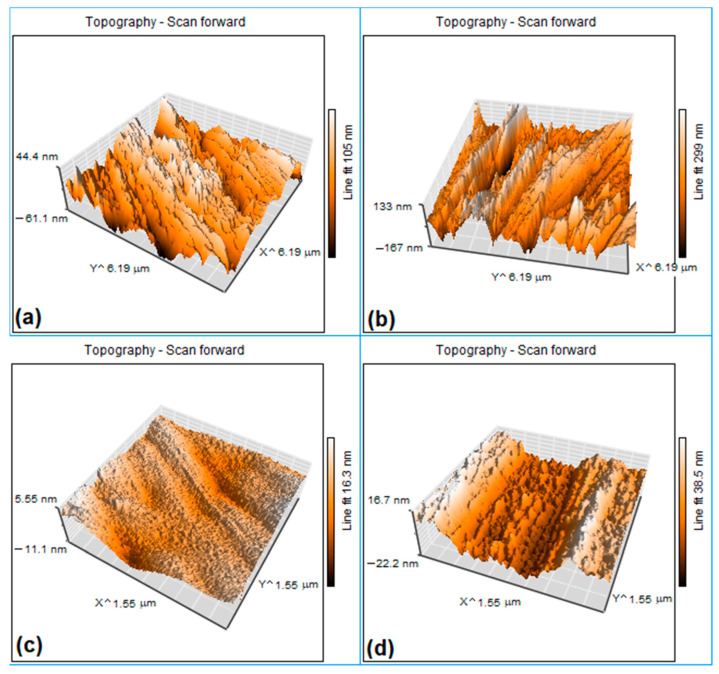
Typical *AFM* micrographs illustrating the morphology of martensite plates after a different number of *FR-SME* training cycles: (**a**) aspect of the convex surface after one cycle; (**b**) aspect of the concave surface after one cycle; (**c**) aspect of the convex surface after sixteen cycles; (**d**) aspect of the concave surface after sixteen cycles.

The representative 3D *AFM* micrographs of fragments cut from outer (convex) and inner (concave) surfaces of the specimens subjected to the first and the sixteenth *FR-SME* training cycles are summarized in Figure 12.

By cumulating the average values of martensite plate widths (measured on surface profile lines) with those of plate heights (determined on *AFM* micrographs), the results listed in Table 5 were obtained.

It must be mentioned that the distinction between B19 and B19′ martensites can hardly be done on *AFM* micrographs [50]. The specimen subjected to one *FR-SME* training cycle has, on its concave surface, an amount of approx. 33% B19′ monoclinic martensite, according to Figure 9d. The typical width of these lathy martensite plates was about 100 nm, according to TEM measurement performed at a Ti_50_Ni_44_Cu_6_ SMA [51].

In our case, according to Table 5, the average martensitic plate dimensions are 152 nm for width and 300 nm for height. By evaluating the evolution of average plate dimensions as an effect of the number of *FR-SME* training cycles, it is noticeable that almost all dimensions markedly decreased, ranging between 13 and 16% of the initial height. The single exception is the average width measured on concave surfaces, which increased from 152 to 200 nm. This diminution of martensite plates, with increasing the number of *FR-SME* training cycles, could be one of the reasons for the reduction of absorbed enthalpy, observed in Figure 5.

## 4. Summary and Conclusions

A Ti_50_Ni_45_Cu_5_ SMA was hot rolled and water quenched, acquiring a structure dominated by B19 orthorhombic martensite but also including B19′ monoclinic martensite. The structural-functional changes caused by *TENS*, *FR-SME,* and *WG-SME* training were investigated by *DSC*, *DMA*, *XRD,* and *AFM*.

By TENS, ultimate stress and strain were determined as 527 MPa and 26%, respectively. After four loading–unloading cycles, energy storage efficiency increased from 12 to 76%, and specific damping capacity decreased from 88 to 24%. As an effect of a permanent strain of 11%, some of the B19 plates were reoriented to B19′ martensite plates.

*FR-SME* training comprised specimen forced forming during one day/cycle. After applying up to 16 cycles to different specimens, a decrease in shape recovery degree was observed from 62% in the first cycle to 33%.

*WG-SME* training was applied to 0.44 g-weighing specimens, which lifted different loads from 100 to 1500 g during heating and lowered them during cooling. As an effect of this procedure, trained specimens were able to develop fully reproducible reversible martensitic transformations, as observed in the specimen trained for 50 cycles under 100g applied load.

*DSC* investigations highlighted the variations of the peak temperature (*A*_50_) and specific absorbed enthalpy (Δ*h_endo_*) as an effect of the training procedures. *FR-SME* training caused a decreasing tendency for both *A*_50_ (from 62 to 58°) and Δ*h_endo_* (from 28.25 to 9.62 J/g in absolute value) when the number of cycles increased from 1 to 16. *WG-SME* revealed a marked stabilization capacity of the thermodynamic character, enabling the occurrence of a temperature memory effect at 60 °C. By high-temperature *DSC*, it was demonstrated that reverse martensitic transformation absorbed more specific enthalpy compared to melting.

*DMA* experiments illustrated a sharp increase in the storage modulus during heating, reaching a rate of 893 MPa/°C, more than four times larger than the values found in the literature. This increase in the storage modulus hardening rate was ascribed to the application of five RT reversible bending cycles by augmenting the amplitude, which intensified the movement of twin boundaries between martensite plate variants, causing internal friction enhancement.

*XRD* patterns revealed the presence of three main metallographic phases, B2 austenite, as well as B19 and B19′ martensites. The presence of stable dominating B19 orthorhombic martensite at RT is unusual at a Ti_50_Ni_45_Cu_5_ SMA. During the first *FR-SME* training cycles, the fraction of B19′ monoclinic martensite significantly increased on behalf of B19. When the number of *FR-SME* increased, the parameters of the B19′ unit cell experienced different variation tendencies. In the case of concave surfaces, the calculated maximum variation rates were +6.59 × 10^−4^ nm/cycle for parameter *a* and −13.28 × 10^−4^ nm/cycle for parameter *c*.

*AFM* micrographs illustrated a marked diminution of martensite plate heights, with 84–87%, as an effect of increasing the number of *FR-SME* training cycles. This reduction may be the cause of the decreased absorbed enthalpy during the reverse martensitic transformation.

## Figures and Tables

**Table 1:** Average deflection angles of the cold (*α^c^*) and hot (*α^h^*) shapes from Figure 2a together with the shape recovery degree (*SRD*).

Cycle No.	*α^c^*, °	*α^h^*, °	*SRD*, %
1	71	139	62.4
2	48.5	130	62
4	55	113	46.4
8	43	111.5	50
16	43.5	89	33.3

**Table 2:** Variations in specific absorbed enthalpy (Δ*h_endo_*) and temperature of reverse transformation peak (*A*_50_) with the number of *FR SME* training cycles according to the average heat flow variation with temperatures from Figure 5d,e.

Cycle No.	Heating Rate °C/min	Δ*h_endo_* J/g	*A*_50_ °C
l	5	−21.65	60.85
	10	−28.25	62.1
2	5	−7.675	63.6
	10	−10.6	65.4
4	5	−11.655	60.7
	10	−25.195	61.3
8	5	−8.17	59
	10	−8.78	60.55
16	5	−9.45	57.5
	10	−9.62	58.05

**Table 3:** Variations of specific absorbed (Δ*h_endo_*) and released (Δ*h_exo_*) enthalpies and temperatures of reverse (*A*_50_) and direct (*M*_50_) transformation peaks with the number of *WG-SME* training cycles according to Figure 6.

Specimen	Δ*h_endo_* J/g	*A*_50_ °C	Δ*h_exo_* J/g	*M*_50_ °C
hot rolled	−29.18	60.4	31.71	37.7
6 cycles	−30.29	47.4	34.71	24.5
50 cycles	−29.28	60.5	37.99	33.5

**Table 4:** Linear fit summary of the experimental values of unit cell parameters from Figure 11.

	*a* (nm)	*b* (nm)	*c* (nm)
*I* (nm)	0.28379 ± 0.00266	0.46041 ± 8.21888 × 10^−4^	0.42281 ± 0.00367
*s* (nm/cycle)	(3.35385 ± 3.23116) × 10^−4^	(6.15111 ± 9.96685) × 10^−5^	(−8.82543 ± 4.45373) × 10^−4^
Residual Sum of Squares	5.01 × 10^−5^	4.77 × 10^−6^	9.52 × 10^−5^
Pearson’s r	0.51404	0.33565	−0.75292
R-Square (COD)	0.26423	0.11266	0.56689
Adj. R-Square	0.01898	−0.18312	0.42252

**Table 5:** Average values of martensite plates according to Figure 12.

	Convex Surface		Concave Surface	
	1st Cycle	16th Cycle	1st Cycle	16th Cycle
Width (nm)	300	265	152	200
Height (nm)	106	17	300	39

## Data Availability

Not applicable.

## References

[1] Buehler W.J., Gilfrich J.V., Wiley R.C. (1963). Effect of Low-Temperature Phase Changes on the Mechanical Properties of Alloys near Composition TiNi. J. Appl. Phys..

[2] Melton K.N., Duerig T.W., Melton K.N., Stockel D., Wayman C.M. (1990). Ni-Ti based shape memory alloys. Engineering Aspects of Shape Memory Alloys.

[3] Matsumoto O., Miyazaki S., Otsuka K., Tamura H. (1987). Crystallography of martensitic transformation in Ti-Ni single crystals. Acta Met..

[4] Wollants P., Roos J.R., Delaey L. (1993). Thermally- and stress-induced thermoelastic martensitic transformations in the reference frame of equilibrium thermodynamics. Prog. Mater. Sci..

[5] Otsuka K., Ren X. (2005). Physical metallurgy of Ti-Ni-based shape memory alloys. Prog. Mater. Sci..

[6] Duerig T.W., Stöckel D., Keeley A., Duerig T.W., Melton K.N., Stockel D., Wayman C.M. (1990). Actuator and work production devices. Engineering Aspects of Shape Memory Alloys.

[7] van Humbeeck J., Stalmans R., Otsuka K., Wayman C.M. (1998). Characteristics of Shape Memory Alloys. Shape Memory Materials.

[8] Furuya Y., Shimada H., Duerig T.W., Melton K.N., Stockel D., Wayman C.M. (1990). Shape memory actuators for robotic applications. Engineering Aspects of Shape Memory Alloys.

[9] Ohkata I., Suzuki Y., Otsuka K., Wayman C.M. (1998). The design of shape memory alloy actuators and their applications. Shape Memory Materials.

[10] Gao Z., Shi Q., Fukuda T., Li C., Huang Q. (2019). An overview of biomimetic robots with animal behaviors. Neurocomputing.

[11] Motzki P., Seelecke S., Olabi A.-G. (2022). Industrial applications for shape memory alloys. Encyclopedia of Smart Materials.

[12] Moberlyt W.J., Melton K.N., Duerig T.W., Melton K.N., Stockel D., Wayman C.M. (1990). Ni-Ti-Cu shape memory alloys. Engineering Aspects of Shape Memory Alloys.

[13] Nam T.H., Saburi T., Shimizu K. (1990). Cu-content dependence of shape memory characteristics in Ti-Ni-Cu alloys. Mater. Trans. JIM.

[14] Wu S.-K., Lin H., Lin T. (2006). Electrical resistivity of Ti–Ni binary and Ti–Ni–X (X = Fe, Cu) ternary shape memory alloys. Mater. Sci. Eng. A.

[15] Harikrishnan K., Chandra K., Misra P.S., Agarwala Vinod S., Šittner P., Heller L., Paidar V. (2009). B19 orthorhombic martensitic transformations in aged TiNiCu shape memory alloys. ESOMAT 2009, Proceedings of the 8th European Symposium on Martensitic Transformations, Prague, Czech Republic, 7–11 September 2009.

[16] Perkins J., Hodson D., Duerig T.W., Melton K.N., Stockel D., Wayman C.M. (1990). The two-way shape memory effect, In Engineering Aspects of Shape Memory Alloys.

[17] Kireeva I.V., Pobedennaya Z.V., Chumlyakov Y.I., Marchenko E.S. (2020). Superelasticity and two-way shape memory effect in [0 0 1]-oriented TiNiCu single crystals under compression. Mater. Lett..

[18] Zhou X., Huang Z., Chen F., Tian B., Li L., Tong Y. (2022). Two-way shape memory effect with excellent cycling stability in TiNiCuNb alloy. Mater. Lett..

[19] Wang Z.G., Zu X.T., You L.P., Feng X.D., Zhang C.F. (2004). Investigation on the two-way shape memory effect and alternating current electrothermal driving characteristics of TiNiCu shape memory alloy. J. Mater. Sci..

[20] Chen L.P., Si N.C. (2008). Influence of thermomechanical training deformations on TWSME in TiNiCu alloy spring. J. Alloys Compd..

[21] Bujoreanu L.-G., Lohan N.M., Pricop B., Cimpoeşu N. (2012). On role of atomic migration in amnesia occurrence during complex thermal cycling of Cu–Zn–Al shape memory alloy. Mater. Sci. Technol..

[22] Vitel G., Paraschiv A.L., Suru M.G., Cimpoesu N., Bujoreanu L.-G. (2011). New calorimetric-structural aspects of temperature memory effect in hot rolled Cu-Zn-Al SMAs. Optoelectron. Adv. Mater.-Rapid Commun..

[23] Ye Z., Li C., Zheng M., Zhang X., Yang X., Gu J. (2022). In situ EBSD/DIC-based investigation of deformation and fracture mechanism in FCC- and L12-structured FeCoNiV high-entropy alloys. Int. J. Plast..

[24] Duerig T.W., Zadno R., Duerig T.W., Melton K.N., Stockel D., Wayman C.M. (1990). An engineer’s perspective of pseudoelasticity. Engineering Aspects of Shape Memory Alloys.

[25] Mihalache E., Pricop B., Comăneci R.I., Suru M.-G., Lohan N.-M., Mocanu M., Ozkal B., Bujoreanu L.-G. (2017). Structural Effects of Thermomechanical Processing on the Static and Dynamic Responses of Powder Metallurgy Fe-Mn-Si Based Shape Memory Alloys. Adv. Sci. Technol..

[26] Mihalache E., Pricop B., Lohan N.-M., Suru M.-G., Ozkal B., Bujoreanu L.-G. (2016). Internal friction evaluation in mechanically alloyed-powder metallurgy Fe-Mn-Si-Cr-Ni shape memory alloys. Int. J. Mod. Manuf. Technol..

[27] Santosh S. (2020). Synthesis, Hot Deformation Behavior and Biocompatibility of Some NiTi-Based Ternary Shape Memory Alloys. Ph.D. Thesis.

[28] Lohan N.M., Pricop B., Bujoreanu L.-G., Cimpoeşu N. (2011). Heating rate effects on reverse martensitic transformation in a Cu-Zn-Al shape memory alloy. Int. J. Mater. Res..

[29] Kajiwara S., Kikuchi T. (1982). Dislocation structures produced by diverse martensitic transformation in a Cu-Zn alloy. Acta Metall..

[30] Liu T.-W., Zheng Y.-J., Cui L.-S. (2015). Transformation Sequence Rule of Martensite Plates and Temperature Memory Effect in Shape Memory Alloys. Acta Met. Sin..

[31] Khangholi S.N., Javidani M., Maltais A., Chen X.-G. (2022). Effect of Ag and Cu addition on the strength and electrical conductivity of Al-Mg-Si alloys using conventional and modified thermomechanical treatments. J. Alloys Compd..

[32] Kireeva I.V., Pobedennaya Z.V., Chumlyakov Y.I., Marchenko E.S. (2021). Effect of stress-induced martensite ageing on the one-way and two-way shape memory effect of [0 1 1]-oriented TiNiCu crystals under tension. Mater. Lett..

[33] Yoshida I., Otsuka K. (2006). Effect of Heat Treatments on the Damping Characteristics of TiNi-Based Shape Memory Alloys. Key Eng. Mater..

[34] Yoshida I., Ono T., Asai M. (2000). Internal friction of Ti–Ni alloy. J. Alloys Compd..

[35] Van Humbeeck J. (2003). Damping capacity of thermoelastic martensite in shape memory alloys. J. Alloys Compd..

[36] Chien C., Wu S.-K., Chang S.-H. (2014). Damping Characteristics of Ti50Ni50−xCux (x = 0~30 at.%) Shape Memory Alloys at a Low Frequency. Materials.

[37] Fabregat-Sanjuan A., Guirado F.G., Ferrando F., De la Flor S. (2018). Identifying the effects of heat treatment temperatures on the Ti50Ni45Cu5 alloy using dynamic mechanical analysis combined with microstructural analysis. Mater. Sci. Eng. A.

[38] Juan J.S., No M.L. (2003). Damping behavior during martensitic transformation in shape memory alloys. J. Alloys Compd..

[39] Chien C., Wu S.-K., Chang S.-H. (2015). Damping Capacities of Ti50Ni50−xCux Shape Memory Alloys Measured under Temperature, Strain, and Frequency Sweeps. Mater. Trans..

[40] Bujoreanu L.-G., Young M.L., Gollerthan S., Somsen C., Eggeler G. (2010). Influence of heat treatment and microstructure on the tensile pseudoelastic response of an Ni-rich NiTi shape memory alloy. Int. J. Mater. Res..

[41] Zhao G., Chen J., Ding C., Fang D., Huang C., Ye X. (2020). Effect of Yttrium on the microstructure, phase transformation and superelasticity of a Ti–Ni–Cu shape memory alloy. Vacuum.

[42] Bumke L., Wolff N., Chluba C., Dankwort T., Kienle L., Quandt E. (2021). Coherent Precipitates as a Condition for Ultra-Low Fatigue in Cu-Rich Ti53.7Ni24.7Cu21.6 Shape Memory Alloys. Shape Mem. Superelasticity.

[43] Sun K., Yi X., Sun B., Gao W., Wang H., Meng X., Cai W., Zhao L. (2019). The effect of Hf on the microstructure, transformation behaviors and the mechanical properties of Ti-Ni-Cu shape memory alloys. J. Alloys Compd..

[44] Haq I.U., Khan M.I., Karim R.A., Raza S.A., Wadood A., Hassan M. (2021). Effect of Various Thermal Treatments and Cu Contents on Precipitation Mechanism, Martensitic Growth, and Cold Workability of Ti50Ni50-xCux Ternary Shape Memory Alloys. J. Eng. Mater. Perform..

[45] Suru M.-G., Paraschiv A.-L., Lohan N.M., Pricop B., Ozkal B., Bujoreanu L.-G. (2014). Loading Mode and Environment Effects on Surface Profile Characteristics of Martensite Plates in Cu-Based SMAs. J. Mater. Eng. Perform..

[46] Suru M.-G., Morosanu C., Bujoreanu L.-G. (2014). Variation tendencies of shape memory alloys surface relief as a function of training-cycling parameters. J. Optoelectron. Adv. Mater..

[47] Jones N., Dye D. (2013). Influence of applied stress on the transformation behaviour and martensite evolution of a Ti-Ni-Cu shape memory alloy. Intermetallics.

[48] Shiva S., Yadaiah N., Palani I., Paul C., Bindra K. (2019). Thermo mechanical analyses and characterizations of TiNiCu shape memory alloy structures developed by laser additive manufacturing. J. Manuf. Process..

[49] Pushin V.G., Kuranova N.N., Pushin A.V. (2016). Structure and mechanical properties of shape-memory alloys of the Ti-Ni-Cu system. Met. Sci. Heat Treat..

[50] Curtis S.M., Wolff N., Dengiz D., Lewitz H., Jetter J., Bumke L., Hayes P., Yarar E., Thormählen L., Kienle L. (2020). Integration of AlN piezoelectric thin films on ultralow fatigue TiNiCu shape memory alloys. J. Mater. Res..

[51] Li J., Yi X., Sun K., Sun B., Gao W., Wang H., Meng X., Song W. (2018). The effect of Zr on the transformation behaviors, microstructure and the mechanical properties of Ti-Ni-Cu shape memory alloys. J. Alloys Compd..

